# Supplementary material for: Impact of Anti-TNFα Treatment on the Humoral Response to the BNT162b2 mRNA COVID-19 Vaccine in Pediatric Inflammatory Bowel Disease Patients
Source: Vaccines (Basel). 2022 Sep 27;10(10):1618. doi: 10.3390/vaccines10101618 (PMC9611127; doi:10.3390/vaccines10101618)
Supplement: Supplementary file 1 [file vaccines-10-01618-s001.zip › vaccines-1922982-supplementary.pdf]

Table S1. Adverse events within 7 days after the first vaccination.

Variable		Overall (*n* = 63)	Patients on Anti-TNFα (*n* = 11)	Patients on Non-Anti-TNFα (*n* = 31)	Patients on 5-ASA (*n* = 21)
Fever	0	60.3 (38)	27.3 (3)	77.4 (24)	52.4 (11)
	1	22.2 (14)	27.3 (3)	9.7 (3)	38.1 (8)
	2	11.1 (7)	27.3 (3)	6.5 (2)	9.5 (2)
	3	0	0	0	0
	4	6.3 (4)	18.2 (2)	6.5 (2)	0
Systemic AEs	Fatigue	38.1 (24)	54.6 (6)	32.5 (10)	38.1 (8)
	Headache	15.9 (10)	36.4 (4)	16.3 (5)	4.8 (1)
	Muscle or joint pain	14.3 (9)	18.2 (2)	13.0 (4)	14.3 (3)
	Nausea	3.2 (2)	0	6.5 (2)	0
	Allergic reaction	0	0	0	0
Severity of systemic AEs	0	60.3 (38)	45.5 (5)	67.7 (21)	57.1 (12)
	1	39.7 (25)	54.6 (6)	32.5 (10)	42.9 (9)
	2	0	0	0	0
	3	0	0	0	0
Local injection site reaction	Pain	81.0 (51)	90.9 (10)	71.0 (22)	90.5 (19)
	Erythema	11.1 (7)	9.1 (1)	13.0 (4)	9.5 (2)
	Swelling	19.0 (12)	18.2 (2)	19.5 (6)	19.0 (4)
Severity of local injection site reaction	0	17.5 (11)	9.1 (1)	26.0 (8)	9.5 (2)
	1	76.2 (48)	90.9 (10)	67.7 (21)	81.0 (17)
	2	4.8 (3)	0	3.2 (1)	9.5 (2)
	3	0	0	0	0

Data are expressed as percentages. TNF = tumor necrosis factor, 5-ASA = 5-aminosalicylic acid, AEs = adverse events. Fever was assessed according to the following scale: 0: none (<37.5 °C), 1: 37.5–38 °C, 2: 38–38.4 °C, 3: 38.5–38.9 °C, and 4: ≥39 °C. Systemic AEs and local injection site reactions were assessed according to the following scale: 0: none; 1: does not interfere with activity; 2: interferes with activity; and 3: prevents daily activity and/or requires hospitalization.

Table S2. Adverse events within 7 days after the second vaccination.

Variable		Overall (*n* = 63)	Patients on Anti-TNFα (*n* = 11)	Patients on Non-Anti-TNFα (*n* = 31)	Patients on 5-ASA (*n* = 21)
Fever	0	50.8 (32)	27.3 (3)	61.3 (19)	47.5 (10)
	1	25.4 (16)	18.2 (2)	19.4 (6)	38.1 (8)
	2	15.9 (10)	36.4 (4)	13.0 (4)	9.5 (2)
	3	1.6 (1)	9.1 (1)	0	0
	4	6.3 (4)	9.1 (1)	6.5 (2)	4.8 (1)
Systemic AEs	Fatigue	49.2 (31)	63.6 (7)	35.5 (11)	61.9 (13)
	Headache	17.5 (11)	36.4 (4)	16.1 (5)	9.6 (2)
	Muscle or joint pain	19.0 (12)	27.3 (3)	16.3 (5)	19.2 (4)
	Nausea	1.6 (1)	0	3.3 (1)	0
	Allergic reaction	0	0	0	0
Severity of systemic AEs	0	47.6 (30)	36.4 (4)	61.3 (19)	33.3 (7)
	1	47.6 (30)	63.6 (7)	32.2 (10)	61.9 (13)
	2	0.8 (2)	0	3.3 (1)	4.8 (1)
	3	0	0	0	0
Local injection site reaction	Pain	79.4 (50)	81.8 (9)	71.0 (22)	90.5 (19)

	Erythema	17.5 (11)	18.2 (2)	19.4 (6)	14.3 (3)
	Swelling	27.0 (17)	27.3 (3)	26.0 (8)	28.6 (6)
Severity of local injection site reaction	0	19.0 (12)	9.1 (1)	29.1 (9)	9.5 (2)
	1	71.4 (45)	81.8 (9)	61.3 (19)	81.0 (17)
	2	7.9 (5)	0	9.7 (3)	9.5 (2)
	3	0	0	0	0

Data are expressed as percentages. TNF=tumor necrosis factor, 5-ASA = 5-aminosalicylic acid, AEs = adverse events. Fever was assessed according to the following scale: 0: none (<37.5 °C), 1: 37.5–38 °C, 2: 38–38.4 °C, 3: 38.5–38.9 °C, and 4: ≥39 °C. Systemic AEs and local injection site reactions were assessed according to the following scale: 0: none; 1: does not interfere with activity; 2: interferes with activity; and 3: prevents daily activity and/or requires hospitalization.

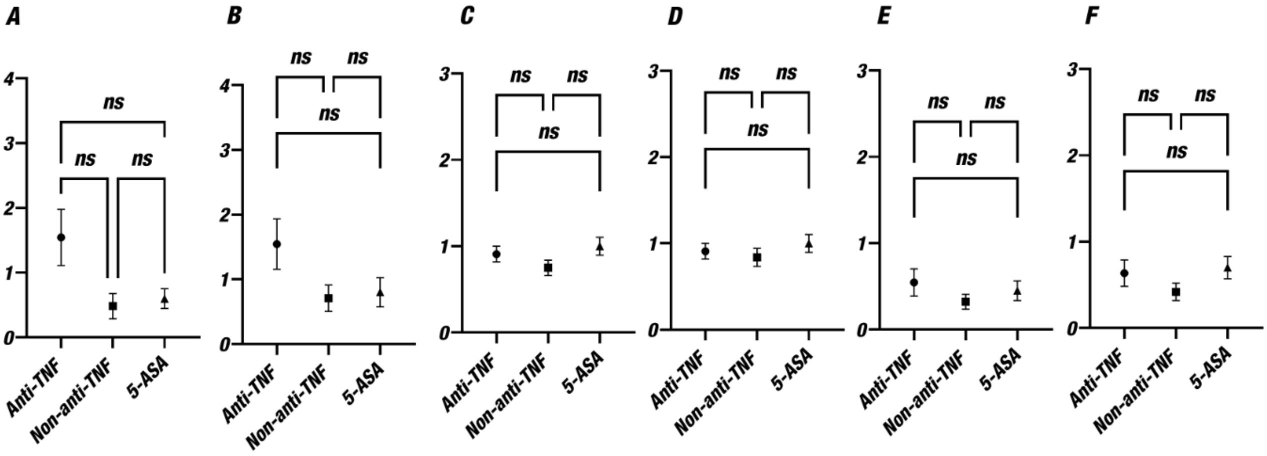

Figure S1. The extent of short-term AEs. The number of patients was 11, 31, 21 in those on anti-TNFα, non-anti-TNFα, and 5-ASA, each. Patients treated with anti-TNFα tended to have higher fevers than other patients, but not significantly higher fevers after the first (**A**) and second (**B**) vaccination. The extent of the local injection site reaction after the first (**C**) and second (**D**) vaccination and the severity of the systemic AEs after the first (**E**) and second (**F**) were comparable in each treatment group. TNF = Tumor necrosis factor, 5-ASA = 5-aminosalicylic acid. Figure represents mean with SEM, and for multiple comparisons, Brown–Forsythe and Welch ANOVA followed Dunnett T3 correction was used, and p-values < 0.05 were considered statistically significant, and ns indicates not significant.
